# Neuromuscular Plasticity: Disentangling Stable and Variable Motor Maps in the Human Sensorimotor Cortex

**DOI:** 10.1155/2016/7365609

**Published:** 2016-08-16

**Authors:** Dominic Kraus, Alireza Gharabaghi

**Affiliations:** Division of Functional and Restorative Neurosurgery and Centre for Integrative Neuroscience, Eberhard Karls University Tuebingen, 72076 Tuebingen, Germany

## Abstract

Motor maps acquired with transcranial magnetic stimulation (TMS) are evolving as a biomarker for monitoring disease progression or the effects of therapeutic interventions. High test-retest reliability of this technique for long observation periods is therefore required to differentiate daily or weekly fluctuations from stable plastic reorganization of corticospinal connectivity. In this study, a novel projection, interpolation, and coregistration technique, which considers the individual gyral anatomy, was applied in healthy subjects for biweekly acquired TMS motor maps over a period of twelve weeks. The intraclass correlation coefficient revealed long-term reliability of motor maps with relevant interhemispheric differences. The sensorimotor cortex and nonprimary motor areas of the dominant hemisphere showed more extended and more stable corticospinal connectivity. Long-term correlations of the MEP amplitudes at each stimulation site revealed mosaic-like clusters of consistent corticospinal excitability. The resting motor threshold, centre of gravity, and mean MEPs across all TMS sites, as highly reliable cortical map parameters, could be disentangled from more variable parameters such as MEP area and volume. Cortical TMS motor maps provide high test-retest reliability for long-term monitoring when analyzed with refined techniques. They may guide restorative interventions which target dormant corticospinal connectivity for neurorehabilitation.

## 1. Introduction

Adaptive reorganization of cortical maps after brain damage is referred to as plasticity and is regarded as relevant during recovery and compensation by reflecting changes of neural circuit architecture and synaptic connectivity [1]. The connectivity of these neuronal networks is, however, also being continuously modified by use-dependent mechanisms independent of any injury or recovery. When studying changes of cortical map plasticity during disease progression or therapeutic interventions, it is therefore necessary to disentangle stable and variable map parameters. In this context, brain stimulation techniques are particularly suitable for monitoring the cortical maps, for example, to probe effective corticospinal connectivity by measuring time-locked motor evoked potentials (MEP) at target muscles. The techniques applied in animal research and human studies, for example, intracortical microstimulation or epicortical electrical stimulation, differ with regard to their level of invasiveness and spatial accuracy [2–7].

Transcranial magnetic stimulation (TMS)—albeit with significantly less spatial resolution than surgical mapping techniques—has been established as a powerful alternative mapping tool for clinical and research application [8]. When applied, for example, in the context of stroke patients, TMS mapping revealed a reduced excitability and a decreased cortical representation of the impaired movement [9, 10]. After short-term therapy, the cortical motor map and the manual dexterity increased at least temporarily [11]. Following longer interventions, clinical gains were paralleled by the recruitment of cortical motor representation in the affected hemisphere outside the primary motor cortex [9, 12, 13].

However, more recent studies have challenged these previous findings by revealing corticospinal connectivity outside the primary motor cortex in healthy subjects [14] as well as by demonstrating relevant variability of the spatial extent of motor maps independent of any intervention [15]. This ambiguity might be related to methodological differences; in recent years, individual magnetic resonance images (MRIs) have been used in conjunction with navigated TMS (nTMS). This technique monitors the coil position, direction, and tilting, thus increasing the repeatability of both coil placement [16, 17] and orientation [18]. When the TMS coil was aligned on the basis of the individual shape of the central sulcus, the somatotopy in the primary motor hand area could be captured [19]. Navigated TMS might thus be more precise than standard TMS, for example, in capturing nonprimary motor cortex corticospinal connectivity [14], but is perhaps still not precise enough to distinguish between the natural daily or weekly fluctuations of the motor map extent [15] and lasting cortical plasticity in the course of a disease or intervention. Such a differentiation would necessitate stable cortical map parameters that are resistant to such natural fluctuations.

In this context, simulation studies have indicated that the individual gyral anatomy has a major impact on TMS-induced electrical field distributions [20–25]. The reliability of motor maps might thus be improved when accounting for interindividual differences in brain anatomy. Combining nTMS maps with individual MRIs facilitated—as a first step on the way—the analysis of group data in normalized space [15, 26, 27]. Previous nTMS approaches, however, still projected the TMS coil positions as a grid of target points on the brain surface, resembling a plane that covered both gyri and sulci, and did not account for differences in cortex morphology [15, 17–19, 28–30]. To overcome this limitation, we recently proposed a novel projection, interpolation, and coregistration technique for estimating nTMS sites onto the individual anatomy, namely, by following the surface curvature of gyri [31]. The novelty of this approach was thus not related to the application of neuronavigation to the TMS mapping procedure itself, as was the case in previous nTMS studies, but instead consisted in the application of the stereotactic information provided by nTMS to visualize the stimulation findings in relation to the specific anatomy [31]. The specific visualization of the stimulation sites, obtained by* nestling* them to the gyral curvature, was complemented by a mathematical interpolation which considered all neighboring stimulation results in a distance-weighted fashion. This technique achieved a lower variability of cortical motor maps between subjects in normalized space than standard TMS mapping [31].

In the present study, we reasoned that this refined TMS technique would also provide high test-retest reliability of cortical motor maps, although the inherent variability of TMS metrics, like other metrics representing human physiology, may be related to many biological reasons. We tested the long-term stability of nTMS in healthy subjects, not for days and weeks as tested previously but for several months, and with six instead of only two or three different measurement time points. Since these previous studies—which applied the standard TMS mapping approach—revealed low retest reliability even for these short observation periods, a repetition of this standard procedure for longer follow-up periods will not provide any further insight. We therefore focused our long-term examination on the novel approach which was recently introduced [31]. Notably, the limited reliability observed in previous studies was not related to focal mapping parameters such as* centre of gravity or hotspot* but to mapping parameters that capture the extent of the cortical motor map, such as the* map area*. We therefore addressed these classical parameters and also applied complementary measures to describe the cortical extent of the cortical motor map, such as motor maps of the* mean spatial overlap, the mean MEP amplitude, and the intraclass correlations of the MEP amplitude* in the present study.

We detected extended sensorimotor areas with high functional overlap between subjects and in the course of the mapping sessions. Therefore, long-term stable map areas could be disentangled from the more fluctuating ones by which they were surrounded. At each stimulation site, intraclass correlations of the MEP amplitudes revealed mosaic-like clusters of consistent corticospinal excitability spanning over distributed areas in the sensorimotor cortex. Moreover, and somewhat unexpectedly, relevant interhemispheric differences with more stable corticospinal connectivity in the nonprimary motor areas of the dominant hemisphere were unraveled, reflecting use-dependent plasticity.

## 2. Material and Methods

### 2.1. Subjects

Twelve right-handed subjects (mean age 24 years, range 19–28, 8 males) with verified right-handedness (EHS > 70) according to the Edinburgh Handedness Inventory [32] were studied in the course of six experiments with a mean of 14.7 days between experiments. In all subjects, cortical motor maps of the nondominant, right hemisphere were captured; in six of the participants, additional motor maps of the dominant, that is, left, hemisphere could be acquired. In three of the subjects, an additional seventh measurement was performed ~1.5 years after the sixth session. All measurements were performed at the same time of day. However, the participants were deliberately not requested to alter their daily routines. We thereby hoped to emulate real-life conditions of clinical practice as closely as possible. All participants gave written informed consent and had no contraindication to TMS [33] or a history of any neurological or psychiatric disease. The studies were approved by the local ethics committee and were in accordance with the declaration of Helsinki.

### 2.2. Mapping Protocol

The cortical mapping was performed by the same person in all experiments (DK) as described previously [31]: we used a navigated TMS stimulator (eXimia®, Nexstim, Helsinki, Finland) and a biphasic figure-8 coil (Nexstim, Helsinki, Finland) with a mean diameter of 50 mm and an estimated focality of 0.68 cm^2^ (eXimia Focal Bipulse, Helsinki, Finland). The neuronavigation system controlled the position, orientation, and tilt angle of the TMS coil. Prior to the mapping, individual anatomical T1-weighted magnetic resonance images were acquired by a 3-Tesla Siemens TIM Trio MRI system (Siemens AG, Erlangen, Germany) using the t1-MPRAGE gradient echo, a field of view (FOV) of 256 mm and 176 sagittal slices, a voxel size of 1 × 1 × 1 mm^3^, a repetition time (TR) of 2300 ms, and an echo time (TE) of 2.98 ms. Individual MRIs were loaded into the eXimia system for coregistration with the subject's head using three anatomical landmarks (nasion + both crux helix) and nine additional points on the scalp (registration error < 2 mm). The electromyography (EMG) signal of the extensor digitorum communis (EDC) of both arms was recorded with the integrated EMG device of the eXimia system (3 kHz sampling rate, band-pass filter of 10–500 Hz) using Ag/AgCl AmbuNeuroline 720 wet gel surface electrodes (Ambu GmbH, Germany). The MEPs were acquired from relaxed muscles. The EDC was chosen for this study, since this muscle is the main target during brain-robot interface-based interventions [34–36] designed for stroke rehabilitation [37, 38]. The electrodes were placed 2 cm apart from each other on the muscle belly of the forearm [39], differently from the procedure usually applied for hand muscles.

For each subject, the cortical representation of the EDC muscle was determined using 40% of maximum stimulator output at the anatomically defined “hand knob” of the primary motor cortex (M1) as the starting position. If the initial stimulator output was not sufficient to elicit MEPs, it was increased in steps of 5%. The current waveform of the stimulator was biphasic. The orientation of the induced current in the brain was posterior-anterior for the first phase and anterior-posterior for the second phase of the stimulus as stipulated by the manufacturer. The orientation of the electric field, calculated on the basis of the individual MRI of each subject by the eXimia software, was kept perpendicular to the central sulcus, and the location with the highest MEP response was selected as the stimulation point. Having determined the “hotspot” with about 30 stimuli by moving the coil around the hand knob, we varied the orientation of the coil within an angle of approximately 90° in steps of roughly 10° and with 3 stimuli at each angle, around the original orientation. Using this method, we were able to ascertain the orientation with the highest response in this spot. This orientation was posterior-anterior in all cases with only slight (±20°) interindividual differences. The resting motor threshold (RMT) was determined using the relative frequency method, that is, selecting the minimum stimulus intensity (by changing the stimulator output in 2% steps of maximum stimulator output (MSO)) that resulted in MEPs >50 *μ*V in the peak-to-peak amplitude in at least 5 out of 10 consecutive trials [40, 41].

The cortical map representation was acquired at 110% RMT with the same coil orientation as was applied at the hotspot. This map was extended in random order around the hotspot with evenly distributed stimuli until MEPs could no longer be evoked in the EDC. Despite some interindividual variability, this procedure was sufficient to cover the entire cortical representation of the EDC in all subjects [31]. A visual grid (5 mm × 5 mm × 5 mm), predefined in the navigation software, was used for guidance during the mapping procedure, applying 2-3 stimuli per cell and resulting in an average of 10 stimuli per 1 cm^2^. Specifically, two stimuli were applied per cell; when one of them did not result in a response, a third stimulus was applied. The actual navigation coordinates of each stimulus were then used for data analysis, resulting in a spacing of approximately 3 mm, due to the small variability of the stimulation sites within each cell. Stimulation sites were visualized on the surface at a depth of 20 mm to ensure that the stimuli were located within the cortex in all subjects (range of scalp to cortex distance: 13–18.5 mm). This procedure was chosen due to the fact that the manufacturer allows adjustments to be made in steps of 5 mm only, that is, at 15 mm, 20 mm, and 25 mm. This TMS protocol thus resulted in stimulation sites 20 mm below the scalp and spaced approximately 3 mm apart with their coordinates located in individual MRI space.

### 2.3. Data Processing

Data were analyzed using Matlab R2010b (MathWorks GmbH, Ismaning, Germany) with a custom-built code, the Toolbox SPM8 (Wellcome Trust Centre for Neuroimaging, London, UK), the FreeSurfer Software Suite (Martinos Centre for Biomedical Imaging, Charlestown, USA), and SPSS V21 (IBM GmbH, Ehningen, Germany).

For data analyses, we then used the actual navigation coordinates (i.e., the MRI coordinates within the reference frame of the eXimia system) of each stimulus, resulting in an interstimulus spacing of approximately 3 mm. Finally, these spots were interpolated for visualization, sampled on a 1 × 1 × 1 mm grid to close the gap between stimulation sites, and then projected onto the gyral anatomy following the procedure described below [31]. Importantly, this interpolation technique increased the reliability of every single stimulus by considering all its neighboring stimulation results in a distance-weighted way. This technique also provides a higher level of focality than the conventional approach of treating each stimulus as a discrete event. The level of focality is thus higher than the actual area activated by the stimulation pulse.

Please note that this interpolation procedure resulted formally in a volume (mm^3^) instead of the conventional surface (mm^2^) to describe the extension of the cortical map. Since the* calculated* value (mm^3^) was proportional to the* real* surface area (mm^2^) and was always calculated in the same way for all sessions, it provided a suitable measure for determining the test-retest reliability. During the mapping, about 100 stimuli were applied, with some subject-to-subject variability due to the individual cortical representation of the EDC [31]. Recent findings indicate that reliable motor maps could be created with around 60 stimuli [42]. During this study, the respective map could also be captured with less than 100 stimuli in subjects who had a small cortical representation of the EDC, while in others, more stimuli were required. Such variability of individual cortical maps has already been shown in detail elsewhere [31]. The procedure lasted for ~15 minutes and the subjects were instructed to keep their muscles relaxed during this time. During offline analysis, the EMG data were visually inspected and any trials in which muscle preactivation was detected were discarded (<1% of all trials had to be removed due to EMG activation).

#### 2.3.1. nTMS Processing

Since the stereotactic information provided by the nTMS (eXimia, Nexstim, Helsinki, Finland) refers to the coil position outside the head only, additional calculations are necessary to translate this information beyond the coil and onto the brain. We therefore used the coordinates of the TMS coil to project all stimulation points of the map onto the cortex in the direction of the magnetic field between the two coil windings [31]. The coil coordinates acquired via the navigation system were thereby transferred to the individual MR image of each subject at a depth of ~20 mm (see previous section).

Thereafter, the mean MEP amplitude and the centre of gravity (CoG) of each map were determined. Due to the uncertainty of the exact stimulation depth using TMS, the CoG is usually calculated in two dimensions only. Moreover, we applied individual space (and not normalized space) to analyze the reliability of the CoG so as to enable us to compare it with the literature. The maximum amplitude-weighted stimulation point was calculated using the following formula [43]:

(1) $$\begin{array}{c} CoG=\frac{\sum a_{i}*x_{i}}{A},\frac{\sum a_{i}*y_{i}}{A} \end{array}$$

with *a*_*i*_ as the MEP amplitude at positions *x*_*i*_ (medial-lateral) and *y*_*i*_ (anterior-posterior) and *A* as the sum of all MEP amplitudes.

The MEP amplitudes of all stimuli were then projected onto a 1 × 1 × 1 mm grid and* interpolated* by taking all neighboring stimulation results into account in a distance-weighted way. This resulted in a three-dimensional map area with* mean MEP amplitude* for each grid cell. The sum of active grid cells (with MEPs > 50 *μ*V) subsequently resulted in the map area and the map volume (area *∗* mean map MEP), that is, the MEP amplitude-weighted area, for each measurement. Please note that this* mean MEP amplitude* is different from the* mean Map MEP amplitude* (Table 2) which captures all the* noninterpolated* stimulation amplitudes of one session.

The individual MRI volumes and coregistered MEP maps were spatially normalized to MNI space, using SPM8 for further group analysis [44].

#### 2.3.2. FreeSurfer Processing

The MNI normalized MRI images were then imported into the FreeSurfer software [31], which aligned the individual central sulci, and a cortical surface structure was reconstructed using the inbuilt functions [45]. An average brain surface with >160*k* mesh points was then created by coregistration of the cortical surface structures [44]. The coregistered MEP maps were first projected onto the individual surface structures with the inbuilt function* mri_vol2surf *of FreeSurfer and then transferred onto the average surface structure with* mri_surf2surf*. As a result, all maps were projected onto the same surface coordinate system, enabling us to gain further statistics for each mesh point of the cortical surface.

This procedure enabled us to calculate the mean MEP amplitude over all measurements and subjects, the mean overlap of all subjects in the course of the experiments (in percent), and the intraclass correlation (ICC) values for the MEP amplitudes at each mesh point.

#### 2.3.3. Statistical Analysis

A repeated measure ANOVA (rmANOVA) with Greenhouse-Geisser correction was performed to determine differences in TMS parameters between sessions. Intraclass correlation was applied to compute the test-retest reliability [46] for mean map MEP, map area, map volume, RMT, coordinates of the CoG, and the MEP amplitudes at each stimulation site, that is, surface mesh point.

A two-way random average measure (ICC(2, *k*)) was chosen in SPSS according to McGraw and Wong [47] for the map parameters. In addition, we calculated an ICC(1, *k*) value for each surface mesh point using the MEP amplitude in that coordinate. ICC values usually range from 0 to 1 but can become negative if the variance in the subject is higher than the group variance. Values above 0.75, between 0.5 and 0.75, and below 0.5 are regarded as reflecting high, moderate, and poor test-retest reliability, respectively [46].

## 3. Results

### 3.1. Group Data of TMS Parameters

**Figure 1:**
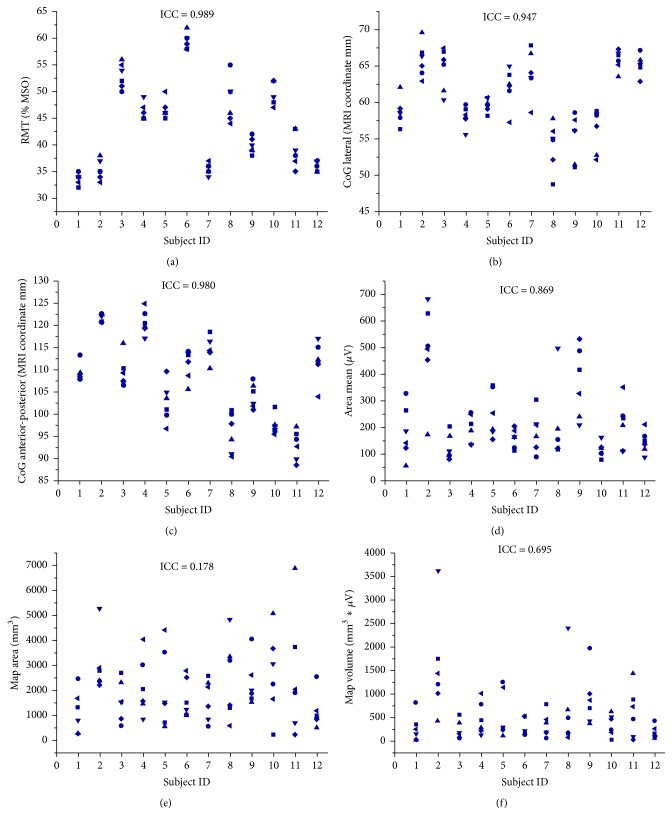
Intrasubject distribution of original data for six experimental sessions (■: Session 1; ●: Session 2; ▲: Session 3; ▼: Session 4; ◆: Session 5; *◄*: Session 6) in the right, nondominant hemisphere of twelve subjects for RMT (a), medial-lateral CoG (b), anterior-posterior CoG (c), mean map MEP amplitude (d), map area (e), and map volume (f).

**Figure 2:**
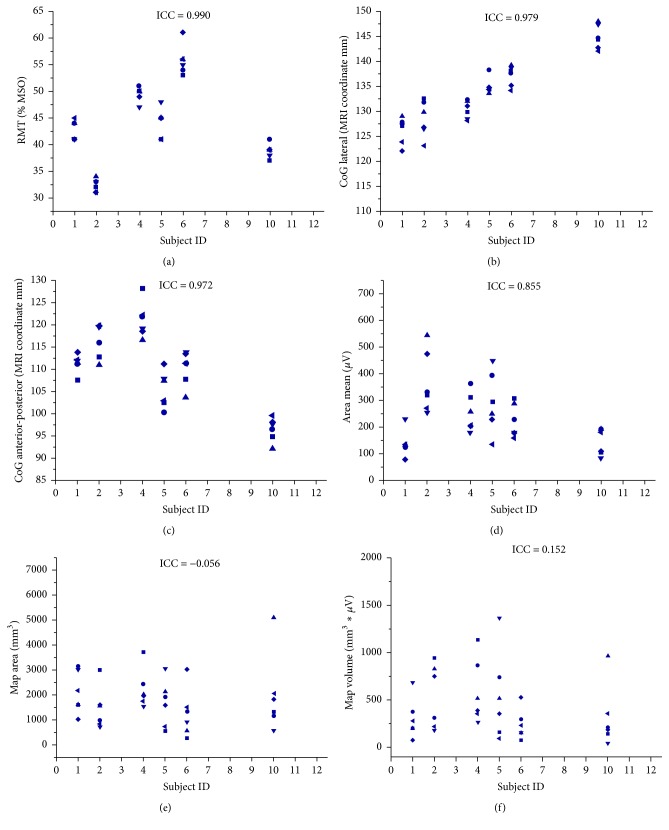
Intrasubject distribution of original data for six experimental sessions (■: Session 1; ●: Session 2; ▲: Session 3; ▼: Session 4; ◆: Session 5; *◄*: Session 6) in the left, dominant hemisphere of six subjects for RMT (a), medial-lateral CoG (b), anterior-posterior CoG (c), mean map MEP amplitude (d), map area (e), and map volume (f).

The data of all experimental sessions was acquired and analyzed without any drop-outs and no significant mean differences of TMS parameters between sessions were revealed in the rmANOVA. The original TMS parameters of each hemisphere are summarized on the group level in Tables 1 and 2 and on the single subject level in Figures 1 and 2, respectively.

### 3.2. Reliability of TMS Parameters

In the nondominant, right hemisphere, ICC values over six sessions showed high reliability for the RMT (ICC = 0.989; 95% Confidence Interval CI: 0.975 to 0.996, Figure 1(a)), the medial-lateral (ICC = 0.947; 95% CI: 0.882 to 0.983, Figure 1(b)) and anterior-posterior CoG (ICC = 0.98: 95% CI: 0.955 to 0.933, Figure 1(c)), and the mean map MEP amplitude, that is, the average of all MEP amplitudes of the cortical map (ICC = 0.869; 95% CI: 0.711 to 0.956, Figure 1(d)). The map volume (ICC = 0.695; 95% CI: 0.32 to 0.899, Figure 1(f)) and map area (ICC = 0.178; 95% CI: −0.879 to 0.73, Figure 1(e)) showed moderate and poor reliability, respectively.

In the dominant, left hemisphere, ICC values over six sessions revealed high reliability for the RMT (ICC = 0.990; 95% CI: 0.970 to 0.998, Figure 2(a)), the medial-lateral (ICC = 0.979; 95% CI: 0.927 to 0.997, Figure 2(b)) and anterior-posterior CoG (ICC = 0.972; 95% CI: 0.914 to 0.996, Figure 2(c)), and the mean map MEP amplitude (ICC = 0.855; 95% CI: 0.566 to 0.977, Figure 2(d)). The map volume (ICC = 0.152; 95% CI: −0.130 to 0.535, Figure 2(f)) and map area (ICC = −0.056; 95%: −0.173 to 0.403, Figure 2(e)) revealed poor reliability.

In three subjects, a seventh session (highlighted in red, Supplementary Figure 1 (a–f) in Supplementary Material available online at http://dx.doi.org/10.1155/2016/7365609) could be acquired for the nondominant hemisphere. The high reliability of the RMT (ICC = 0.995; 95% CI: 0.976 to 1), medial-lateral CoG (ICC = 0.973; 95% CI: 0.878 to 0.999) and anterior-posterior CoG (ICC = 0.892; 95% CI: 0.537 to 0.997), and the mean map MEP amplitude (ICC = 0.928; 95% CI: 0.664 to 0.998) in the previous six sessions could be preserved in the seventh measurement, that is, ~1.5 years after the sixth session.

### 3.3. Motor Map Group Data

**Figure 3:**
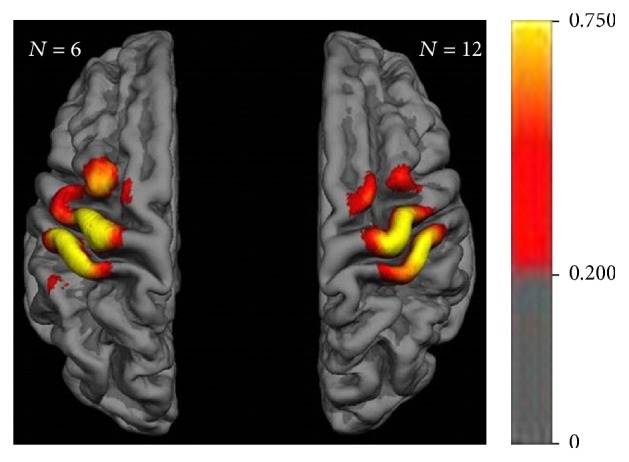
Motor map group data of individual means over time with mean overlap percentage. Color bar indicates percent of subjects presenting with MEPs > 50 *μ*V throughout the experimental sessions.

The mean overlap percentage revealed a high spatial overlap over the hand area of M1 and the corresponding somatotopic sensory (S1) area of both hemispheres; that is, in these regions (indicated in yellow) at least 75% of the subjects presented with MEPs > 50 *μ*V. This core area was surrounded by a fringe area (indicated in red) extending medially and laterally on M1 and S1 and anteriorly on the premotor (PM) cortex. In this fringe area, less than 75% of the subjects presented with MEPs > 50 *μ*V (Figure 3).

**Figure 4:**
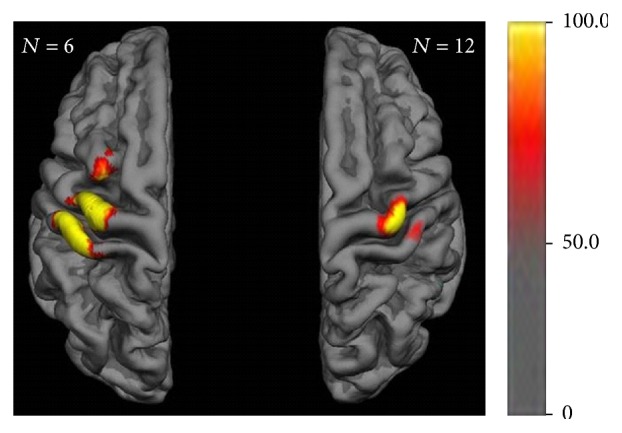
Motor map group data of individual means over time with mean MEP amplitude. Color bar indicates mean MEP amplitude in *μ*V throughout the experimental sessions.

The mean MEP amplitude depicted a smaller activation area than the previous overlap map; that is, activation was confined to those cortical areas in which all subjects had mean MEPs > 100 *μ*V (indicated in yellow) and >50 *μ*V (indicated in red) (Figure 4). Notably, this area covered a large part of M1 and S1 and extended towards the PM cortex in the left, dominant hemisphere, while it remained fairly restricted to the hand knob of M1 and the corresponding S1 in the right, nondominant hemisphere. These interhemispheric differences remained stable, even when the right cortical map was restricted to the very same six subjects who were analyzed for the left cortical map (Supplementary Figure 2).

### 3.4. Motor Map Reliability

**Figure 5:**
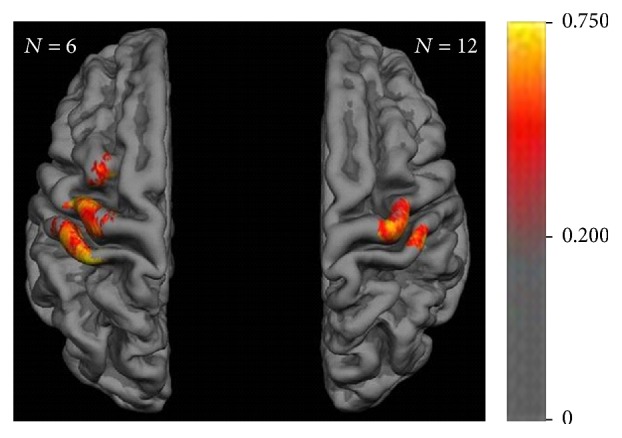
Motor map reliability with intraclass correlation (ICC) for the MEP amplitudes > 50 *μ*V at each mesh point. Color bar indicates ICC value of repeatability in the course of the six experiments revealing mosaic-like clusters of consistent corticospinal excitability.

The intraclass correlation (ICC) values for the MEP amplitudes at each mesh point confirmed the previous cortical maps (of the mean MEP amplitude), showing the same interhemispheric differences and revealing moderate to high reliability (up to >0.75) of the MEP amplitude in the course of the six experiments (Figure 5). Interestingly, these long-term correlations of the MEP amplitude at each stimulation site presented mosaic-like clusters of consistent corticospinal excitability spanning over distributed areas in the sensorimotor cortex.

## 4. Discussion

This study introduces complementary and highly consistent measures for capturing the extent of the cortical motor map with transcranial magnetic stimulation (TMS) and demonstrates the high test-retest reliability of these maps for long observation periods by considering the individual gyral anatomy. We examined motor-evoked potentials (MEPs) of the extensor digitorum communis muscle of healthy subjects over a period of twelve weeks with six biweekly acquired TMS motor maps, whereas previous studies on TMS test-retest reliability spanned observation periods of one to six weeks with a total of two to three measurements only [15, 48–54]. The demonstrated consistency of the acquired motor map parameters over several months qualifies them as biomarkers for monitoring disease progression or the effects of therapeutic interventions, for example, in the context of neurorehabilitation. However, these results need to be extrapolated carefully to individuals with brain damage since patients might have more variable cortical physiology. Particular attention should be paid to the specific TMS parameters chosen for long-term monitoring. Like previous studies, but for longer observation periods, we were able to disentangle the highly stable TMS parameters, that is, the resting motor threshold (RMT), centre of gravity (CoG), and mean MEPs across all TMS sites, from the more variable ones, that is, the map area. We, therefore, suggest not transferring the classical motor map parameters,* map area* and* volume*, to patients but rather the complementary ones introduced and tested in this study, that is, motor maps of the* mean spatial overlap*, the* mean MEP amplitude*, and the* intraclass correlations of the MEP amplitude* (see paragraphs below).

More specifically, the high reliability, captured by the intraclass correlation (ICC), of the RMT and mean map MEP amplitude confirmed previous findings following shorter observation periods [49–53]. Former findings on the consistency of the CoG were more variable [15, 48, 51, 53] than the high reliability in the present study for observation periods of up to 1.5 years.

When it came to the cortical representation area of corticospinal connectivity, the findings were more variable. With regard to the classical parameter* map area*, this study demonstrated poor reliability in the course of six sessions. This finding agrees with previous observations of decreasing reliability of the map area from moderate/high [51, 52] to poor/moderate [15] when increasing the length of the observation period and the number of measurements from two to three. These findings are probably related to the individual conditions of the subjects over time, that is, reflecting the natural daily or weekly fluctuations of the motor map extent [15]. To differentiate them from lasting cortical plasticity in the course of a disease or intervention, more stable cortical map parameters that are resistant to such natural fluctuations would be necessary.

Accordingly, complementary measures for capturing the extent of the cortical motor map were suggested in the present study and revealed spatially specific areas of high reliability throughout the whole observation period of twelve weeks: motor maps of the mean spatial overlap, the mean MEP amplitude, and the ICC of the MEP amplitude enabled us to disentangle a highly reliable core from the surrounding fringe areas of corticospinal connectivity. Future studies may test whether the demonstrated reliability of these complementary motor map parameters will persist when acquired with fixed coil positions (e.g., lateromedial, posteroanterior) or monophasic stimulation.

The overlap map of the present study revealed a core over the hand area of M1 and S1, surrounded by less consistent findings that extended medially and laterally on the sensorimotor cortex and anteriorly on the premotor cortex (Figure 3). These observations were confirmed by the two other motor maps, that is, maps of the mean MEP amplitude and the ICC of the MEP amplitude. However, both of these covered a smaller cortical area than the overlap map. Notably, the maps of the mean (Figure 4) and ICC (Figure 5) of the MEP amplitude in particular revealed relevant interhemispheric differences. In the left, dominant hemisphere, these maps covered a large area of M1 and S1 and extended towards the PM cortex, whereas they remained fairly restricted to the hand knob of M1 and the corresponding S1 in the right, nondominant hemisphere. Moreover, the ICC map unraveled mosaic-like clusters of consistent corticospinal excitability spanning over distributed areas in the sensorimotor cortex and intermingling with spots of decreased reliability.

We interpret the spatial differences between the overlap maps and the mean MEP amplitude maps as a reflection of the high variability of the classical TMS parameter* map area*. More specifically, we propose that the map area represents the instantaneous cortical representation, that is, the natural daily or weekly fluctuations of the motor map extent, and that the mean MEP amplitude map (Figure 4) reflects a stable motor map that is more resistant to this variability.

Rapid functional plasticity of the map area has already been described during different learning processes. Comparing implicit versus explicit motor learning could show an increase of the motor map during the implicit learning period, which was reversed to baseline as soon as explicit knowledge was gained [55]. In another study with Braille readers, the cortical map area varied with the activity of the hand, that is, showing a larger map area during working days than at weekends [56].

By contrast, the stable interhemispheric differences of the mean MEP amplitude map and the ICC map in this study were very probably related to the right-handedness of the participants. This implied a lifelong higher use of the right hand in activities of daily living and therefore a persistent use-dependent reorganization and more extended (towards premotor and somatosensory areas) cortical representation area of this hand in the left, dominant hemisphere [54, 57]. However, further studies with more subjects are necessary to draw definite conclusions.

The present study confirmed earlier animal experiments [58–61] and human studies [14, 39, 62–64], which indicated that corticospinal connections are not limited to the primary motor cortex but extend to different regions of the sensorimotor system. Approximately half of the primate brain's pyramidal tract neurons are located in postcentral areas, for example, the primary somatosensory cortex, sharing functional properties with regard to movement-related activity and discharge patterns as a function of muscle strength with precentral pyramidal tract neurons [31, 65–67]. In the present study, we confirmed this extended corticospinal connectivity of the somatosensory cortex and demonstrated marked interhemispheric differences, that is, highly reliable MEPs elicited from the left S1 of the dominant hemisphere, in healthy subjects. However, due to the rather nonfocal nature of TMS, a complementary explanation of these findings might be possible. Even if the centre of the TMS coil is over the primary somatosensory cortex, this does not necessarily mean that somatosensory cortex stimulation produces the descending volley. It could mean that neurons located rather posterior in the motor cortex, but still anterior to the somatosensory cortex, are activated by the magnetic stimulation delivered to S1 [39]. Therefore, we clearly acknowledge that it is not possible for this type of study to draw conclusions regarding the precise site of cortical stimulation. On the other hand, intraoperative electrical stimulation in humans with both mono- and bipolar focal stimulation of the premotor and somatosensory cortex also elicited MEPs [39, 63], supporting the hypothesis of direct corticospinal connectivity of nonprimary motor cortex areas.

Despite the fact that they have considerably less spatial resolution than surgical mapping techniques, the TMS maps unraveled mosaic-like clusters of consistent corticospinal excitability. This is consistent with the findings of intracortical microstimulation in nonhuman primates which demonstrated that identical movements are elicited by the stimulation of multiple and noncontiguous sites [39, 60]. Although previous studies have already suggested that TMS maps are suitable for reproducing these experimental microstimulation findings in humans [15, 52], the present examination is the first to demonstrate the long-term reliability of this specific cortical pattern and to characterize the extended topographic distribution in the sensorimotor cortex intermingled with spots of decreased reliability. We consider this pattern to be evidence of the specific activation of neuronal pools in the respective cortical areas, for example, S1 or PM, thus rendering the alternative explanation, that is, the current spread to distant areas such as to M1 and the pyramidal tract, rather unlikely. These findings therefore underline the TMS technique presented here as a powerful and precise mapping tool for clinical and research application.

Interestingly, this study is the first to demonstrate the long-term retest reliability of corticospinal connectivity of the premotor cortex, for the left, dominant hemisphere in particular. The right, nondominant hemisphere showed a larger fluctuation of the PM corticospinal connectivity, suggesting that this pathway is a dormant reserve for compensatory activation, for example, when the nondominant hand is used more frequently or when lesions of the M1 corticospinal connections, for example, after stroke, necessitate alternative pathways. Along the same lines, recent neurofeedback interventions have explored the plasticity of the nondominant, right hemisphere in the healthy [39] and lesioned brain [37, 68]. These findings indicate that combining motor imagery-related *β*-band event-related desynchronization with proprioceptive feedback in a brain-robot interface environment [69, 70] might be sufficient to unmask latent corticospinal connectivity [37], redistribute sensorimotor connectivity patterns, and enhance corticospinal pathways of both the S1 and PM cortex [39, 71]. Moreover, pilot data applying this concept demonstrated operant conditioning of the targeted brain state and provided a direct brain-behavior relationship [72] with functional gains after stroke, which were specific for the trained task [68].

## 5. Conclusion

We demonstrated the high test-retest reliability of the applied TMS mapping technique for long observation periods. This study revealed the long-term reliability of motor maps with relevant interhemispheric differences, that is, more extended and stable corticospinal connectivity in the sensorimotor cortex and nonprimary motor areas of the left, dominant hemisphere. Different cortical maps allowed the disentangling of stable cortical reorganization from more rapid plastic fluctuations. Mosaic-like clusters of consistent corticospinal excitability spanning over distributed areas in the sensorimotor cortex indicated functionally specific and spatially precise activation of neuronal pools by TMS. Moreover, these findings may guide restorative interventions addressing dormant corticospinal connectivity for neurorehabilitation.

## Supplementary Material

Supplementary figure 1: Intrasubject distribution of original data for seven experimental sessions. Supplementary figure 2: Motor map group data with mean MEP amplitude in the right hemisphere for the same six subjects examined in the left hemisphere.

## Figures and Tables

**Table 1:** Right hemisphere: original group data of TMS parameters (resting motor threshold, the coordinates of the centre of gravity, mean map MEP amplitude, map area, and map volume) for six experimental sessions in the right, nondominant hemisphere of twelve subjects.

	Session					
	Session 1	Session 2	Session 3	Session 4	Session 5	Session 6
RMT (% MSO)	43.1 ± 8.0	44.1 ± 8.6	44.2 ± 8.8	44.1 ± 8.4	43.0 ± 8.2	43.1 ± 8.4
CoG m-l (mm)	60.7 ± 6.4	61.3 ± 3.8	60.9 ± 5.4	60.9 ± 4.3	60.7 ± 4.6	60.0 ± 4.4
CoG a-p (mm)	109.0 ± 8.4	108.9 ± 9.7	108.0 ± 8.9	107.3 ± 10.6	107.2 ± 9.6	105.7 ± 10.9
Mean map MEP amplitude (*µ*V)	255.6 ± 155.3	240.7 ± 147.9	165.3 ± 48.3	227.5 ± 177.7	190.9 ± 144.7	227.5 ± 118.1
Map area (mm^3^)	1795.6 ± 1006	2291.4 ± 1118.3	2306.8 ± 1979.8	1958.4 ± 1597.6	1522.7 ± 969.4	2299.5 ± 1125.5
Map volume (mm^3^ *∗µ*V)	512772.6 ± 475433.5	650398.9 ± 579946.1	405527.8 ± 380330.5	678479.5 ± 1120122.4	331512.3 ± 345943.7	579769.2 ± 444784.6

Mean ± SD.

**Table 2:** Left hemisphere: original group data of TMS parameters (resting motor threshold, the coordinates of the centre of gravity, mean map MEP amplitude, map area, and map volume) for six experimental sessions in the left, dominant hemisphere of six subjects.

	Session					
	Session 1	Session 2	Session 3	Session 4	Session 5	Session 6
RMT (% MSO)	42.3 ± 7.9	44.7 ± 7.4	44.7 ± 7.8	43.7 ± 7.9	44.3 ± 10.2	43.7 ± 8.7
CoG m-l (mm)	134.5 ± 6.2	135.5 ± 5.9	135.3 ± 7.2	133.9 ± 8.2	132.2 ± 7.2	131.0 ± 7.3
CoG a-p (mm)	108.9 ± 11.3	109.4 ± 9.5	106.9 ± 8.5	111.7 ± 8.2	112.4 ± 7.7	111.3 ± 8.9
Mean map MEP amplitude (*µ*V)	242.5 ± 98.9	269.6 ± 106.8	275.5 ± 142.7	227.9 ± 122.7	209.5 ± 140.9	179.4 ± 53.1
Map area (mm^3^)	1726.8 ± 1359.7	1805 ± 839.8	2134.0 ± 1530.9	1616.3 ± 1132.3	1818.5 ± 667.4	1491.5 ± 614.9
Map volume (mm^3^ *∗µ*V)	442868.3 ± 470689.2	468965.8 ± 268648.9	531075.8 ± 324023.0	448895.9 ± 498086.2	381524.2 ± 239264.6	258593.3 ± 100338.3

Mean ± SD.
