# Supplementary material for: Neuromuscular Plasticity: Disentangling Stable and Variable Motor Maps in the Human Sensorimotor Cortex
Source: Neural Plast. 2016 Aug 16;2016:7365609. doi: 10.1155/2016/7365609 (PMC5004060; doi:10.1155/2016/7365609)

Supplementary figure 1: Intrasubject distribution of original data for seven experimental sessions (■: Session 1; ●: Session 2; ▲: Session 3; ▼: Session 4; ♦: Session 5; ◄: Session 6; ◄: Session 7) in the right, non-dominant hemisphere of three subjects for RMT (a), medial-lateral CoG (b), anterior-posterior CoG (c), mean map MEP amplitude (d), map area (e) and map volume (f).This figure shows the findings for the subjects 3, 9 and 12 of Figure 1 including the data of the seventh measurement (acquired ~1.5 years after session 6).

a)

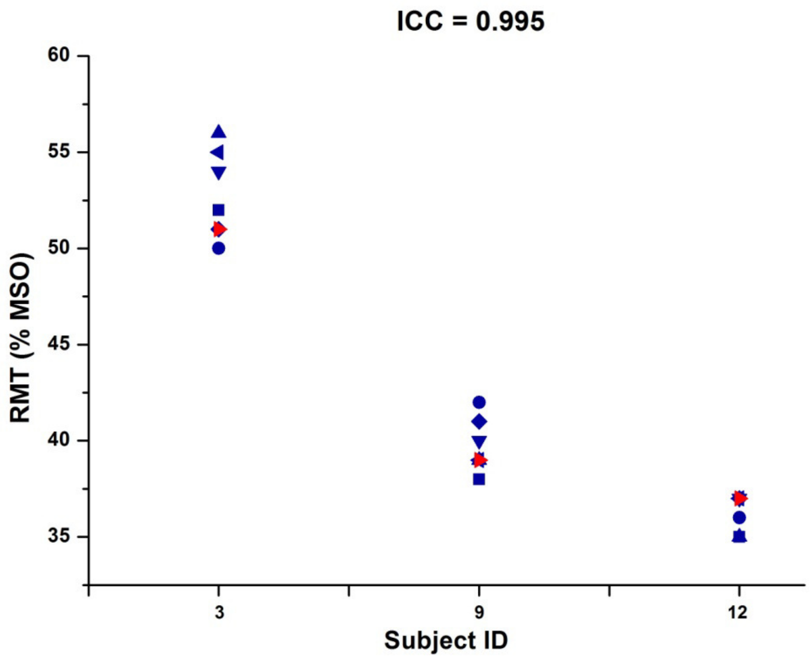

b)

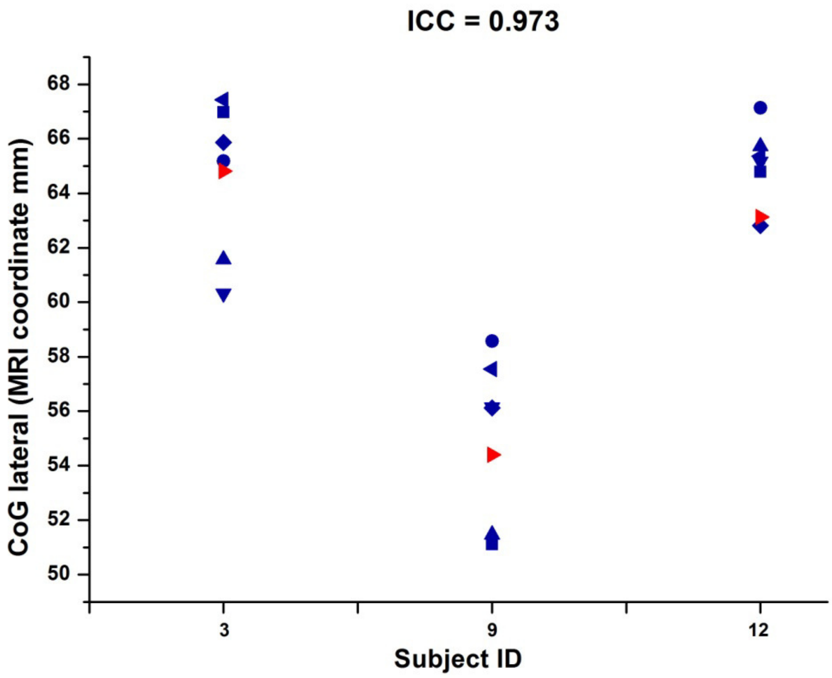
ICC = 0.973
68
66
64
62
60
58
56
54
52
50
CoG lateral (MRI coordinate mm)
3
9
12
Subject ID

c)

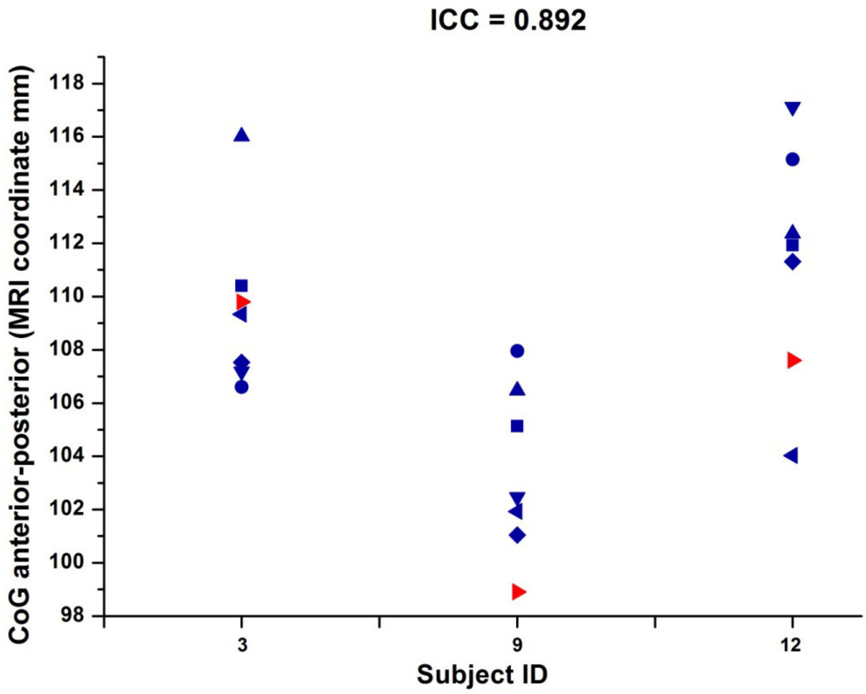
ICC = 0.892
118
116
114
112
110
108
106
104
102
100
98
CoG anterior-posterior (MRI coordinate mm)
3
9
12
Subject ID

d)

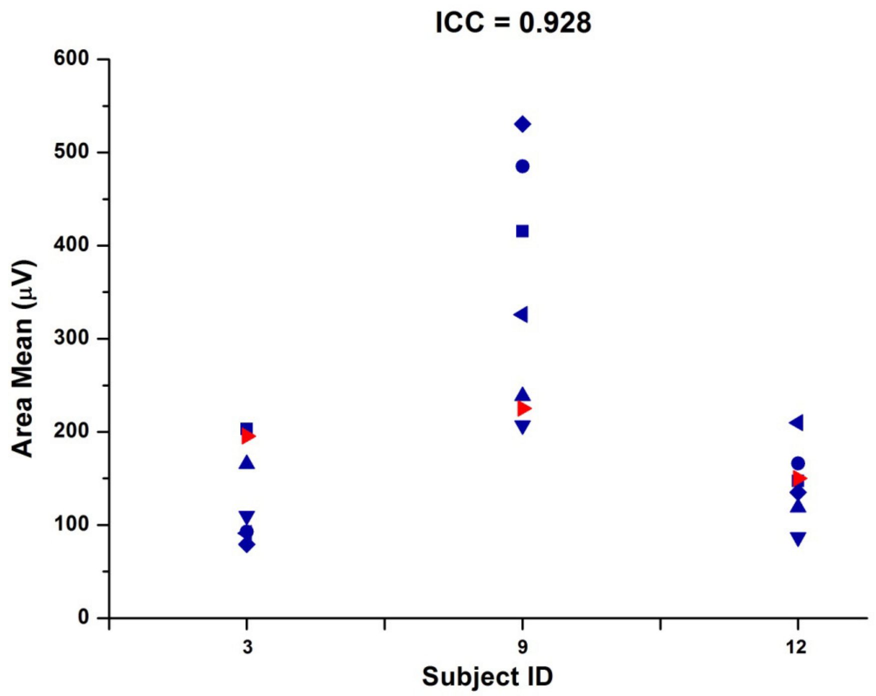

ICC = 0.928
Area Mean (μV)
0
100
200
300
400
500
600
3
9
12
Subject ID

e)

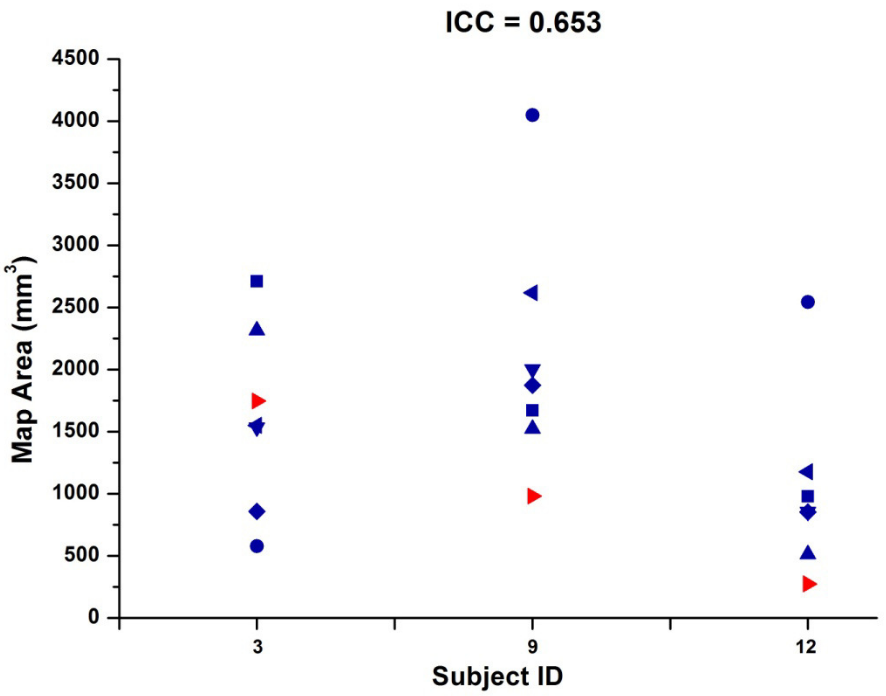

ICC = 0.653
Map Area (mm3)
0
500
1000
1500
2000
2500
3000
3500
4000
4500
3
9
12
Subject ID

f)

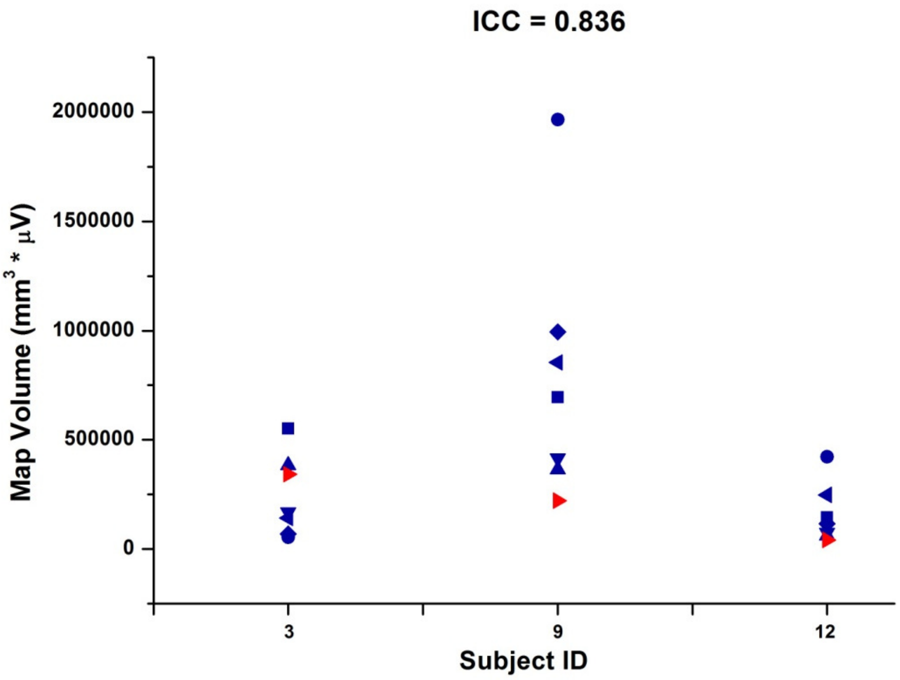

Supplementary figure 2: Motor map group data with mean MEP amplitude in the right hemisphere for the same six subjects examined in the left hemisphere. Color bar indicates mean MEP amplitude in µV throughout the experimental sessions.

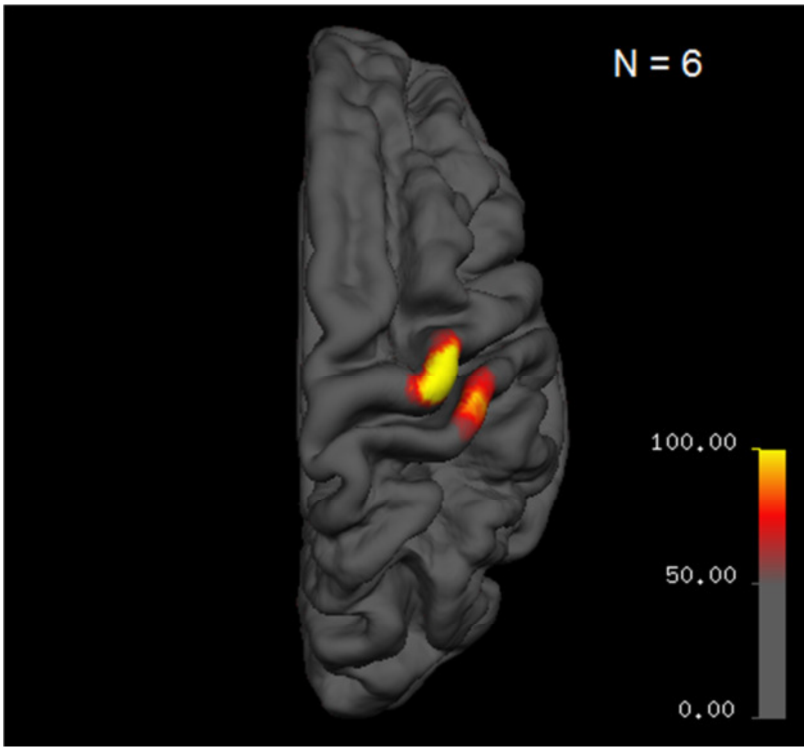

Supplement: Supplementary file 1 — Supplementary figure 1: Intrasubject distribution of original data for seven experimental sessions. Supplementary figure 2: Motor map group data with mean MEP amplitude in the right hemisphere for the same six subjects examined in the left hemisphere. [file 7365609.f1.pdf]
